# Molecular insights into lower respiratory tract microbiota reveal disease-specific biomarkers and shared microbial networks in asthma and COPD

**DOI:** 10.3389/fcimb.2026.1840378

**Published:** 2026-07-21

**Authors:** Yuanping Huang, Yanfei Zhao, Xue Xin, Shan Lin

**Affiliations:** 1 Department of Respiratory Medicine, The First Hospital of Jilin University, Changchun, Jilin, China; 2 Department of Pediatrics, The First Hospital of Jilin University, Changchun, Jilin, China

**Keywords:** asthma, biomarker, co-occurrence, COPD, infection, lung function, machine learning, microbiology

## Abstract

**Background:**

Lower respiratory tract infections (LRTIs) exacerbate chronic airway diseases, yet phenotype-specific microbial signatures are poorly defined. We applied broncho-alveolar lavage fluid (BALF) genomic sequencing to identify biomarkers for asthma and chronic obstructive pulmonary disease (COPD).

**Methods:**

Between December 2023 and February 2025, 1–146 adults with suspected LRTI enrolled from the First Hospital of Jilin University underwent BALF next-generation sequencing. Patients were stratified by lung function, with the impaired pulmonary function group further divided into asthma, COPD-mild-moderate, and COPD-severe subgroups. Disease-specific key biomarkers were identified using machine learning algorithms and analyzed for co-occurrence.

**Results:**

Impaired pulmonary function was not only associated with pathogenic microorganisms and its higher microbial burden, but also associated with a distinct community structure. Random forest models revealed disease-specific biomarkers, with *Prevotella intermedia*, *Finegoldia magna*, and Human parvovirus enriched in asthma, *Veillonella parvula*, Human respiratory syncytial virus, and *Haemophilus influenzae* enriched in COPD-mild-moderate, and Human respiratory syncytial virus, Human coronavirus, and Human parainfluenza virus enriched in COPD-severe. Co-occurrence network identified hubs linking asthma-centric (*Haemophilus parainfluenzae* and *Schaalia odontolytica*) and COPD-centric (*Klebsiella pneumoniae*, *Veillonella parvula*, and *Streptococcus constellatus*) clusters, suggesting potential cross-phenotype microbial crosstalk.

**Conclusions:**

Genomic sequencing profiling delineates distinct yet overlapping airway microbiota across separate pulmonary dysfunctional diseases - asthma and COPD. Compact biomarker panels classify each condition accurately and reveal shared microbial hubs that may drive chronic inflammation and exacerbations, supporting microbiome-guided precision diagnostics and therapy.

## 1 Introduction

Lower respiratory tract infections (LRTIs) are a leading cause of morbidity and mortality worldwide, especially among individuals with chronic respiratory diseases such as asthma and chronic obstructive pulmonary disease (COPD) (Troeger et al., 2018; Feldman and Shaddock, 2019; Niederman and Torres, 2022). Patients with impaired pulmonary function are particularly susceptible to recurrent or severe infections, often accompanied by complex and dynamic changes in the airway microbiota (Frayman et al., 2017; Kussek et al., 2022; Yan et al., 2022). Increasing evidence suggests that the composition and structure of the respiratory microbiome play critical roles in disease susceptibility, exacerbation risk, and clinical outcomes (Wang et al., 2016; Mayhew et al., 2018; Dickson et al., 2020; Dicker et al., 2021). However, the microbial profiles associated with distinct types of pulmonary dysfunction remain poorly characterized.

Next-generation sequencing (NGS) technologies have emerged as powerful tools for identifying a broad spectrum of pathogenic microorganisms in clinical samples, offering enhanced sensitivity (Gu et al., 2019). This approach is especially valuable in patients with LRTIs, where conventional diagnostic techniques often fail to identify causative pathogens, particularly in polymicrobial or atypical infections (Miao et al., 2018; Blauwkamp et al., 2019; Gu et al., 2020). Moreover, understanding the microbial differences between patients with preserved versus impaired pulmonary function - and among distinct phenotypes such as asthma and COPD - may offer novel diagnostic and prognostic biomarkers and enhance our understanding of microbial contributions to disease heterogeneity (Liu et al., 2020; Su et al., 2022; Yu et al., 2022).

In this study, we used NGS to comprehensively profile the lower airway microbiota in patients with suspected LRTIs, stratified by pulmonary function and clinical diagnosis. Our aim was to identify disease-specific microbial signatures and to explore potential microbiological links between asthma and COPD. These findings may offer new perspectives on microbial biomarkers for respiratory disease classification and contribute to a better understanding of the microbiological underpinnings of pulmonary dysfunction.

## 2 Methods

### 2.1 Study design and participants

**Figure 1:**
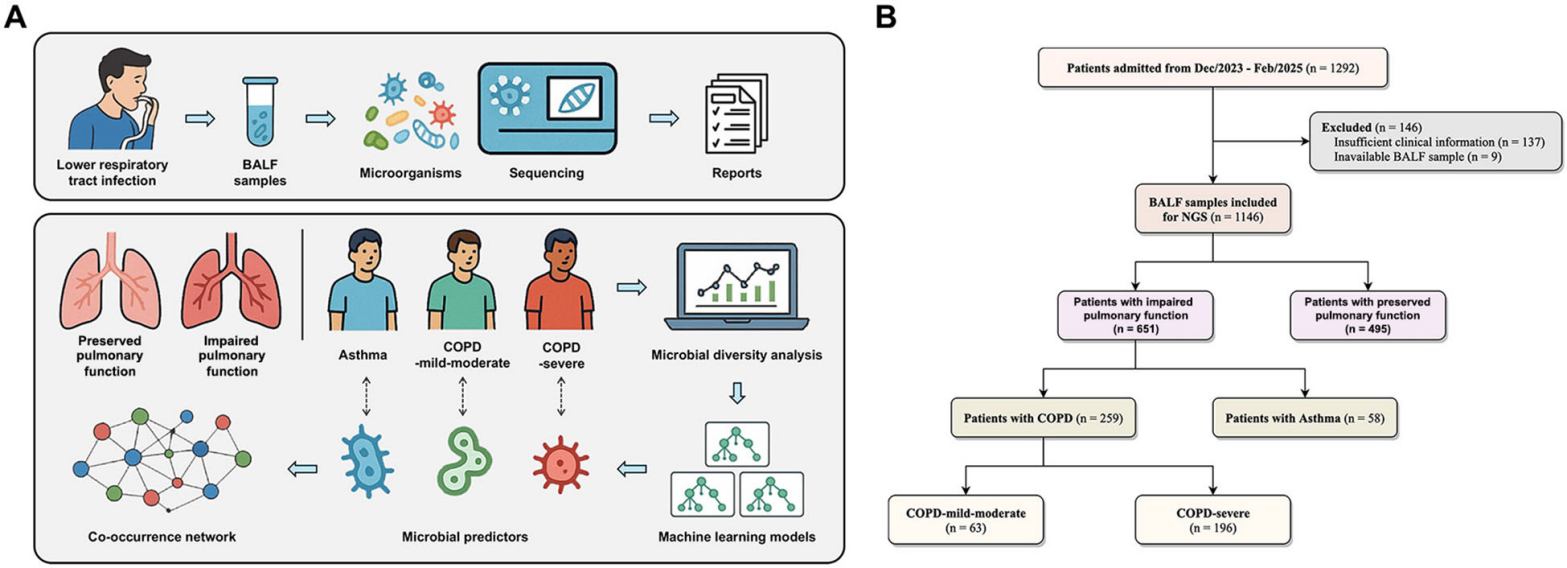
Study design and participants. **(A)** Workflow of the study. BALF, bronchoalveolar lavage fluid; COPD, chronic obstructive pulmonary disease. **(B)** Workflow of the patient enrollment. NGS, next-generation sequencing.

This study was conducted at the Department of Respiratory Medicine, the First Hospital of Jilin University (**Figure 1A**). Patients with suspected respiratory infection who were admitted to our department between December 2023 and February 2025 were consecutively included. Inclusion criteria encompassed (1) suspected lower respiratory infection (defined as a new-onset radiological findings on chest images or a hematologic parameter abnormality combined with at least one compatible symptom, such as fever, cough, asthma, or dyspnea); (2) confirmed clinical diagnosis of lung function (preserved pulmonary function or impaired pulmonary function) and chronic respiratory diseases (asthma and COPD). Specifically, impaired pulmonary function was defined as meeting any of the following pulmonary function test (PFT) criteria: FEV1/FVC < 0.7, FVC% predicted < 80%, FEV1% predicted < 80%, or DLCO% predicted < 80%. Patients who did not meet any of these criteria were classified as having preserved pulmonary function; (3) sufficient bronchoalveolar lavage fluid (BALF) samples for the NGS and conventional microbiological tests (CMTs, including BALF culture, polymerase chain reaction [PCR], and serological tests). Patients with insufficient clinical information or inavailable BALF sample were excluded.

This study design received approval from the Ethics Committee of the First Hospital of Jilin University in accordance with the Declaration of Helsinki (2025-396). The study was conducted with the consent of every human participant.

### 2.2 Sample collection and analysis

Bronchoalveolar lavage was performed on all enrolled participants, and fluid from the middle segment was collected as the study sample. All BALF samples underwent CMT, and remaining aliquots were preserved for NGS. Specifically, the aliquots were transferred into sterile, RNase/DNase-free cryotubes and immediately stored at −80 °C until nucleic acid extraction.

### 2.3 Clinical data collection

Detailed demographic and clinical data were extracted from electronic medical records, including age, gender, immune status (immunocompetent, immunocompromised by cancer chemotherapy, and immunocompromised by diabetes), symptom (cough, fever, and dyspnea), smoking history, sequential organ failure assessment (SOFA) score, and hematologic parameters (white blood cell [WBC], neutrophil [NE], lymphocyte [LYM], C-reactive protein [CRP], and procalcitonin [PCT]).

### 2.4 Next-generation sequencing

Before extracting the nucleic acid, host DNA was first depleted from BALF samples using the MolYsisTM Basic 5 Kit (Molzym GmbH & Co. KG, Bremen, Germany). Total nucleic acids (inclusive of both DNA and RNA) were then extracted using the Magnetic Pathogen DNA/RNA Kit (Tiangen Biotech [Beijing] Co., Ltd, Beijing, China). To enable the simultaneous clinical detection of both DNA microbes and RNA viruses within a single sequencing run, the RNA portion was reverse-transcribed into cDNA using the Hieff NGS Double Stranded cDNA Synthesis Kit (Yeasen, Shanghai, China) without prior DNA depletion. This generated a combined DNA/cDNA pool for subsequent library construction. The concentration of the total DNA pool was quantified using the QubitTM Double-Stranded DNA High Sensitivity Assay Kit (Thermo Fisher Scientific Inc., Waltham, MA, USA). Then DNA libraries were prepared using the VAHTS Universal Plus DNA Library Prep Kit for MGI (Vazyme, Nanjing, China) with an initial input of 2 ng. Meanwhile, library quality control was performed with the Agilent 2100 Bioanalyzer (Agilent Technologies Inc., Santa Clara, CA, USA) to evaluate DNA concentration and fragment size distribution. Libraries exhibiting a dominant fragment peak between 240 bp and 350 bp and a concentration exceeding 1 ng/μL were deemed qualified. Approved libraries were pooled, denatured, and circularized to form single-stranded DNA circles. DNA nanoballs (DNBs) were subsequently generated through rolling circle amplification. The resulting DNBs were loaded onto sequencing chips and subjected to single-end 50-bp sequencing on the BGISEQ-500 (BGI, Shenzhen, China) platform, yielding approximately 10 to 20 million raw reads per library, which is an established adequate sequencing depth for clinical pathogen detection (mNGS) using 50-bp single-end sequencing. To monitor and mitigate the impact of environmental and reagent-derived contaminants (the ‘kitome’), no-template controls (NTCs, utilizing sterile nuclease-free water) were routinely processed and sequenced in parallel with clinical samples in each batch.

Following sequencing, low-quality reads, short reads, and adapter were removed using Fastp (version 0.23.4). A strict bioinformatic decontamination strategy was then applied: microorganisms identified in the BALF samples were cross-referenced against the background microbial profile of their corresponding batch NTCs. Taxa with an abundance in the sample that did not significantly exceed that in the NTC (defined as a sample-to-NTC strict mapping read ratio of < 10) were flagged as background contaminants and filtered from downstream analysis, which is particularly critical for samples with lower microbial biomass. After strict bioinformatic depletion of human-derived reads (described below), the retained high-quality microbial reads (median approximately 100,000 reads per sample, as reflected in Figure X) were utilized for downstream analysis. Clean reads were aligned to three human reference genomes (hg38, T2T-CHM13, and YH1) using Burrows-Wheeler Aligner (BWA, version 0.7.17-r1188). Human-derived reads were subsequently excluded using Samtools (version 1.6). Then the remaining reads were aligned against a custom microbial database using BWA to derive annotations (**Supplementary Table 1**). After that, the annotation results were further validated by BLAST (version 2.12.0) to ensure accuracy.

### 2.5 Clinical diagnosis

Asthma was diagnosed in accordance with the criteria outlined in the 2025 Global Initiative for Asthma (GINA) strategy report (GINA, 2025). COPD was identified in patients presenting with acute exacerbations secondary to LRTIs. In line with the guidelines established by the American Thoracic Society (ATS) and the European Respiratory Society (ERS) (Evensen, 2010), COPD exacerbations were stratified by severity based on the required healthcare utilization. Specifically, mild exacerbations were defined as those requiring treatment with short-acting bronchodilators (SABDs) only, and moderate exacerbations as those requiring treatment with SABDs plus antibiotics and/or oral corticosteroids; patients meeting either of these criteria were collectively classified as the COPD-mild-moderate group. Conversely, severe exacerbations were defined as those requiring hospitalization or a visit to the emergency department, and these patients were categorized as the COPD-severe group. Following the aforementioned bioinformatic background subtraction, the remaining microorganisms identified by NGS were independently evaluated by a minimum of three clinicians holding at least an associate senior professional title, and classified into four levels, including causative pathogen, possibly causative pathogen, microorganism without pathogenic role, and not causative pathogen (Fourgeaud et al., 2023). The clinical diagnosis was established by clinicians following a comprehensive assessment that integrated patient age, gender, immune status, medical history, symptoms, hematologic parameters, radiological findings, CMT results, prognosis, and other relevant clinical data (**Supplementary Figure 1**).

### 2.6 Machine learning model development

To identify key microbial features discriminating between distinct clinical phenotypes, machine learning models employing the random forest algorithm were developed using the scikit-learn library in Python. Three independent binary classification models were constructed, with the target definitions set as follows: (1) asthma versus non-asthma among patients with impaired pulmonary function; (2) COPD versus non-COPD among patients with impaired pulmonary function; and (3) COPD-severe versus COPD-mild-moderate within the COPD subgroup. The input features consisted of the normalized read counts of the identified microorganisms. The models were trained and validated using a bootstrap sampling strategy to ensure robustness. During the training phase, hyperparameter optimization (including the number of estimators and maximum depth) was performed to maximize the area under the receiver operating characteristic curve (AUC). The model’s discriminative performance was subsequently evaluated on the bootstrap-derived test set. Feature importance was extracted based on the Gini impurity reduction (weight) to rank the most robust microbial predictors. In the context of NGS data, the absence of a specific microbial taxon in a sample was biologically interpreted as an abundance below the detection limit; thus, missing microbial feature values were imputed as zero. For clinical variables, patients with missing critical clinical data or insufficient BALF samples were excluded prior to the analysis, as predefined in our inclusion/exclusion criteria.

### 2.7 Statistical analysis

For descriptive statistics, continuous variables were presented as medians and interquartile ranges (IQR), whereas categorical variables were reported as frequencies and percentages. Data normality was assessed by the Shapiro-Wilk test. Nonparametric variables were compared using Mann-Whitney test. All tests were two-tailed and significance threshold was set at p value ≤ 0.05. All statistical analyses were performed using SPSS Statistics (version 26.0, IBM Corp., Armonk, NY, USA). All figures were drawn using R software (version 4.3.1, R Foundation for Statistical Computing, Vienna, Austria), Python (version 3.11, Python Software Foundation, Wilmington, DE, USA), and GraphPad Prism (version 9.5.0, GraphPad Software LLC., San Diego, CA, USA).

## 3 Results

### 3.1 Characteristics of participants in this study

Between December 2023 and February 2025, 1–292 patients admitted for evaluation of lower respiratory tract infection were screened for inclusion (**Figure 1B**). Of these, 146 patients (11.3%, 146/1292) were excluded due to insufficient clinical information or unavailable BALF sample. The remaining 1–146 patients had BALF samples collected for NGS-based microbiological profiling. Lung function testing classified 495 patients (43.2%, 495/1146) as preserved pulmonary function group and 651 patients (56.8%, 651/1146) as impaired pulmonary function group. The impaired pulmonary function group was further divided by clinical diagnosis: 58 patients (8.9%, 58/651) met diagnostic criteria for asthma and 259 patients (39.8%, 259/651) for COPD. Within the COPD subgroup, 63 patients (24.3%, 63/259) were experiencing an COPD-severe, while 196 patients (75.7%, 196/259) were in a mild to moderate phase.

Baseline demographic characteristics were shown in **Table 1**. Among the 1–146 patients enrolled (median age 66 years, IQR 55-73), 21.3% were immunocompromised (16.1% with diabetes, 5.2% with cancer). The predominant symptoms were cough (73.4%), fever (39.4%) and dyspnea (35.9%), with a median SOFA score of 1 and modest elevations in inflammatory markers (median CRP 45.75 mg/L, PCT 0.12 ng/mL). Overall, patients with impaired pulmonary function were similar to the entire cohort in age, immune status, smoking history, and clinical values, but demonstrated a higher proportion of dyspnea (45.6% vs. 35.9%). Within this subgroup, patients with asthma were youngest (median 63 years, IQR 50-73), showed a higher proportion of immunocompromised (25.9%), most frequently presented with cough (86.2%), and had the lowest inflammatory markers (median CRP 17.80 mg/L, PCT 0.08 ng/mL). COPD-mild-moderate patients were older (median 70 years, IQR 66-78), most frequently presented with dyspnea (76.1%), and had longer smoking histories (median 10 years, IQR 0-38) and higher SOFA scores (median 2, IQR 1-2). Those with COPD-severe were oldest (median 72 years, IQR 66-81) and exhibited the greatest dyspnea prevalence (81.0%) and SOFA scores (median 2, IQR 2-3).

**Table 1:** Demographic and clinical characteristics of patients in this study.

Index	Overall (n = 1146)	Impaired pulmonary function (n = 651)	Asthma (n = 58)	COPD-mild-moderate (n = 259)	COPD-severe (n = 63)
Gender					
Female, n (%)	505 (44.1%)	297 (45.6%)	28 (48.3%)	126 (48.6%)	33 (52.4%)
Male, n (%)	641 (55.9%)	354 (54.4%)	30 (51.7%)	133 (51.4%)	30 (47.6%)
Age (years), median (IQR)	66 (55-73)	67 (58-74)	63 (50-73)	70 (66-78)	72 (66-81)
Immune status					
Immunocompetent, n (%)	901 (78.6%)	512 (78.6%)	43 (74.1%)	213 (82.2%)	55 (87.3%)
Diabetes, n (%)	185 (16.1%)	104 (16.0%)	11 (19.0%)	35 (13.5%)	8 (12.7%)
Cancer, n (%)	60 (5.2%)	35 (5.4%)	4 (6.9%)	11 (4.2%)	0 (0%)
Symptoms					
Fever, n (%)	452 (39.4%)	237 (36.4%)	21 (36.2%)	69 (26.6%)	16 (25.4%)
Cough, n (%)	841 (73.4%)	470 (72.2%)	50 (86.2%)	203 (78.4%)	49 (77.8%)
Dyspnea, n (%)	411 (35.9%)	297 (45.6%)	24 (41.4%)	197 (76.1%)	51 (81.0%)
Smoking (years), median (IQR)	0 (0-30)	0 (0-30)	0 (0-10)	10 (0-38)	10 (0-30)
SOFA score, median (IQR)	1 (0-2)	1 (1-2)	1 (0-2)	2 (1-2)	2 (2-3)
Laboratory tests					
WBC (10^9/L), median (IQR)	9.20 (5.52-13.15)	9.31 (5.51-13.11)	9.08 (5.55-12.32)	10.30 (7.31-13.45)	11.14 (7.89-14.67)
NE (%), median (IQR)	79.75 (65.10-89.10)	80.30 (66.55-89.55)	81.40 (63.30-91.20)	82.70 (71.13-90.08)	88.45 (80.25-91.50)
LYM (%), median (IQR)	11.95 (6.00-20.93)	11.40 (5.65-19.90)	11.40 (5.60-22.10)	10.05 (5.30-16.38)	6.80 (4.75-11.00)
CRP (mg/L), median (IQR)	45.75 (9.73-109.95)	45.70 (9.70-108.80)	17.80 (4.80-67.90)	44.90 (10.05-121.93)	33.60 (11.63-110.70)
PCT (ng/mL), median (IQR)	0.12 (0.06-0.41)	0.11 (0.05-0.42)	0.08 (0.04-0.15)	0.11 (0.05-0.35)	0.11 (0.05-0.40)

COPD, chronic obstructive pulmonary disease; IQR, interquartile range; SOFA, sequential organ failure assessment; WBC, white blood cell; NE, neutrophils; LYM, lymphocyte; CRP, C-reactive protein; PCT, procalcitonin.

### 3.2 Microbiological characteristics of pulmonary impairment

**Figure 2:**
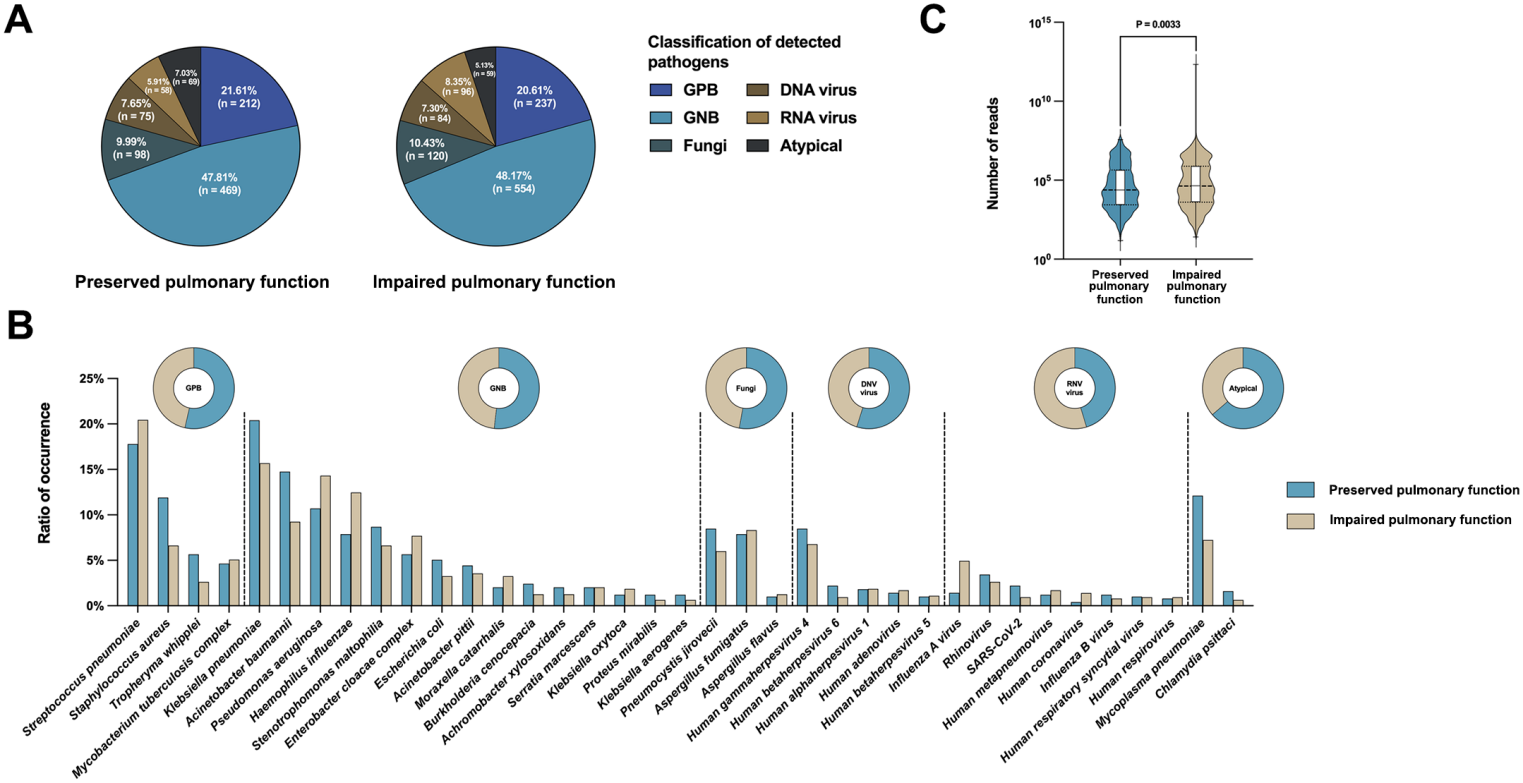
Pathogens in infected patients with good and impaired pulmonary function. **(A)** Percentage of types of pathogens in the two groups. GPB, gram-positive bacteria; GNB, gram-negative bacteria. **(B)** Ratio of occurrence of pathogens in the two groups. **(C)** Number of reads of pathogens detected by next-generation sequencing (NGS) in the two groups. The p value was generated by the Mann-Whitney test, with less than 0.05 indicating a statistically significant difference.

In this study, NGS identified numerous species of microorganisms. (**Supplementary Figure 2**). Among patients with impaired pulmonary function, 85.56% were diagnosed with causative or possibly causative pathogens, a proportion similar to those in patients with preserved pulmonary function (84.64%; **Supplementary Figure 3**). At the pathogen-group level, both cohorts were dominated by gram-negative bacteria, which accounted for 47.81% of detected organisms in the preserved pulmonary function group and 48.17% in the impaired pulmonary function group (**Figure 2A**). Gram-positive bacteria were the second most abundant category (21.61% vs. 20.61%), followed by fungi (9.99% vs. 10.43%). RNA viruses and atypical pathogens were the main differences between the two groups, with RNA viruses being more frequent in the impaired pulmonary function group (8.35% vs. 5.91%) and atypical pathogens being more frequent in the preserved pulmonary function group (7.03% vs. 5.13%).

At the species level, *Streptococcus pneumoniae* was the most commonly identified pathogen overall, occurring in 20.43% of impaired pulmonary function samples versus 17.78% of preserved pulmonary function samples (**Figure 2B**). In contrast, key nosocomial gram-negative species such as *Pseudomonas aeruginosa* and *Acinetobacter baumannii* showed higher prevalence in the preserved pulmonary function group (20.40% and 14.75%, respectively) than in the impaired pulmonary function group (15.67% and 9.22%). Among fungal pathogens, *Pneumocystis jirovecii* was detected more frequently in patients with preserved pulmonary function (8.48% vs. 5.99%), while *Aspergillus fumigatus* showed an opposite trend (7.88% vs. 8.29%). Viral and atypical pathogens, including Human gammaherpesvirus 4 (8.48% vs. 6.76%), Rhinovirus (3.43% vs. 2.61%), SARS-CoV-2 (2.22% vs. 0.92%), and *Mycoplasma pneumoniae* (12.12% vs. 7.22%), were also modestly enriched in the preserved pulmonary function cohort. Furthermore, quantification of total NGS reads per sample revealed a significantly greater microbial burden in infected patients with impaired pulmonary function (p = 0.0033; **Figure 2C**). Specifically, this difference mainly exhibited in *Haemophilus influenzae* (p = 0.0342), *Serratia marcescens* (p = 0.0099), Human betaherpesvirus 6 (p = 0.0031), and Human respiratory syncytial virus (p = 0.0173), among others (**Supplementary Figure 4**).

Principal coordinates analysis encompassing both pathogenic and non-pathogenic microorganisms revealed a marked divergence in microbial composition between patients with good versus impaired pulmonary function (p = 0.002; **Supplementary Figure 5**). These findings underscore a potential association between lung function status and the overall microbial community structure.

### 3.3 Specific biomarkers associated with asthma and COPD

To delineate the specific microbial community structure associated with distinct pulmonary dysfunctions, further analyses were conducted.

**Figure 3:**
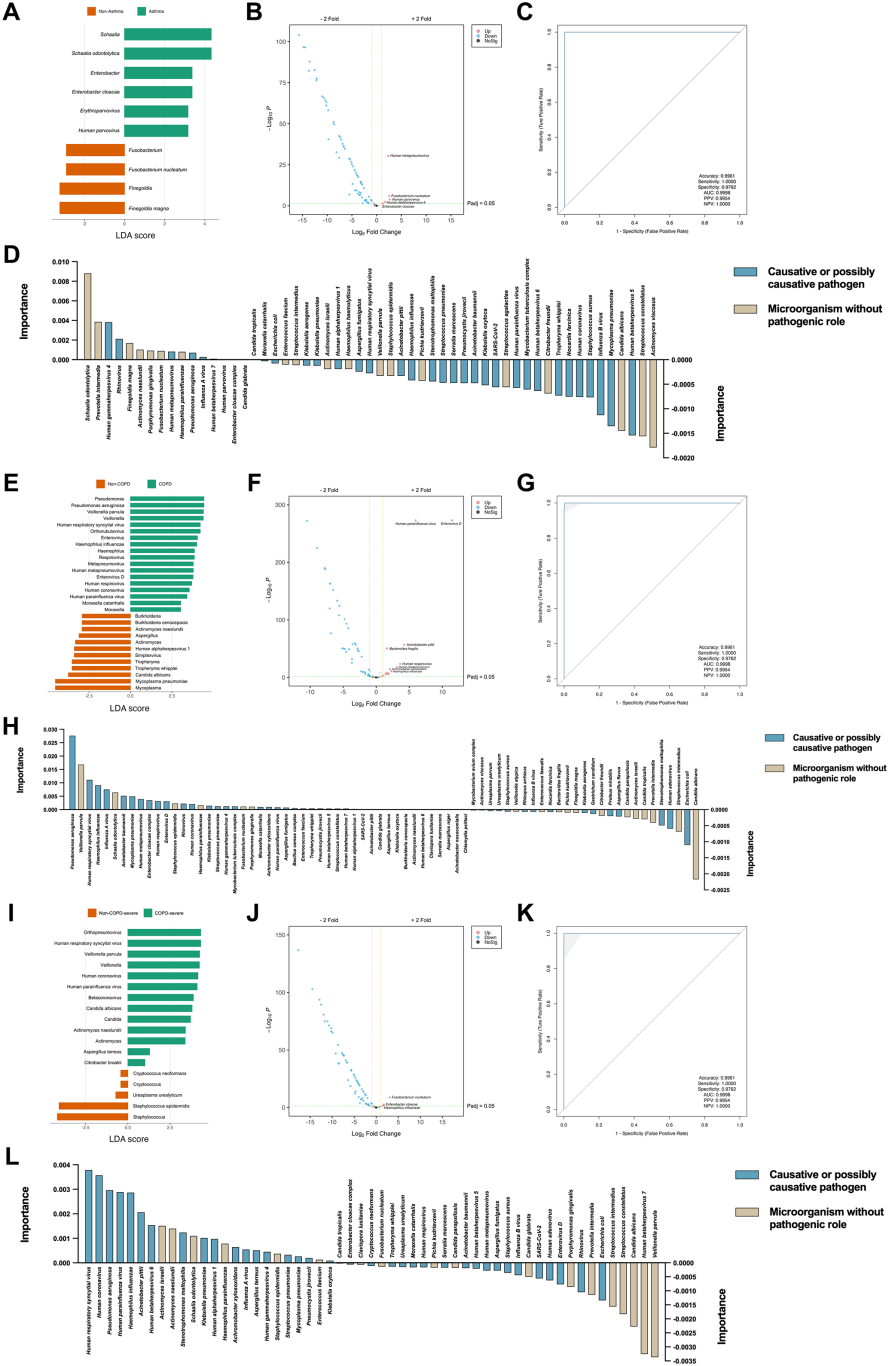
Analysis of infection biomarkers in chronic respiratory diseases. **(A)** Linear discriminant analysis (LDA) of microorganisms identified by next-generation sequencing (NGS) in infected patients with and without asthma. **(B)** DESeq2 analysis of microorganisms identified by NGS in infected patients with and without asthma. **(C)** Performance of the random forest model in bootstrap test set. AUC, area under the receiver operating characteristic curve; PPV, positive predictive value; NPV, negative predictive value. **(D)** Importance of characteristics extracted from the random forest model. Cyan color represents pathogenic microorganisms, and brown color represents non-pathogenic microorganisms. **(E)** LDA of microorganisms identified by NGS in infected patients with and without chronic obstructive pulmonary disease (COPD). **(F)** DESeq2 analysis of microorganisms identified by NGS in infected patients with and without COPD. **(G)** Performance of the random forest model in bootstrap test set. **(H)** Importance of characteristics extracted from the random forest model. Cyan color represents pathogenic microorganisms, and brown color represents non-pathogenic microorganisms. **(I)** LDA of microorganisms identified by NGS in infected patients with and without COPD-severe. **(J)** DESeq2 analysis of microorganisms identified by NGS in infected patients with and without COPD-severe. **(K)** Performance of the random forest model in bootstrap test set. **(L)** Importance of characteristics extracted from the random forest model. Cyan color represents pathogenic microorganisms, and brown color represents non-pathogenic microorganisms.

Among patients with and without asthma, initial microbiological assessments demonstrated significant compositional differences. Linear discriminant analysis effect size (LEfSe) identified 6 taxa significantly enriched in patients with asthma, including *Schaalia* spp., *Enterobacter* spp., and Erythroparvovirus (**Figure 3A**). Differential abundance analysis using DESeq2 revealed significant upregulation of Human metapneumovirus in the asthma cohort (**Figure 3B**). To further identify the key microbial features discriminating between asthmatic and non-asthmatic groups, a machine learning model employing the random forest algorithm was developed. Following hyperparameter optimization, the model demonstrated near-perfect discriminative performance on a bootstrap-derived test set (AUC = 0.9998) (**Supplementary Figure 6A**; **Figure 3C**). Feature importance analysis revealed *Schaalia odontolytica* (weight = 0.0088) as the most robust positive predictor of asthma status, followed by *Prevotella intermedia* (weight = 0.0039; **Figure 3D**). Among pathogenic microorganisms, Human gammaherpesvirus 4 (weight = 0.0038) emerged as the strongest positive predictor of asthma.

In the COPD cohort versus non-COPD subjects, LEfSe detected 18 taxa enriched in COPD patients, including *Pseudomonas* spp. and *Veillonella* spp. (**Figure 3E**). DESeq2 analysis showed significant upregulation of *Acinetobacter pittii* and *Bacteroides fragilis* in COPD (**Figure 3F**). The optimized random forest model yielded an AUC of 0.9998 on the bootstrap test set (**Supplementary Figure 6B**; **Figure 3G**). Importance metrics ranked *Pseudomonas aeruginosa* (weight = 0.0276) as the most robust predictor of COPD status, followed by *Veillonella parvula* (weight = 0.0168, non-pathogenic microorganism), Human respiratory syncytial virus (weight = 0.0111), and *Haemophilus influenzae* (weight = 0.0091; **Figure 3H**).

Among patients with COPD-severe, LEfSe identified 13 taxa enriched during exacerbation, including *Orthopneumovirus* spp., Human respiratory syncytial virus, and *Veillonella* spp. (**Figure 3I**). DESeq2 revealed significant overrepresentation of *Fusobacterium nucleatum* in the COPD-severe group (**Figure 3J**). The corresponding random forest classifier achieved an AUC of 0.9998 following optimization (**Supplementary Figure 6C**; **Figure 3K**). Feature importance analysis highlighted Human respiratory syncytial virus (weight = 0.0038) as the top predictor of exacerbation, with Human coronavirus (weight = 0.0036), *Pseudomonas aeruginosa* (weight = 0.0030), Human parainfluenza virus (weight = 0.0029), and *Haemophilus influenzae* (weight = 0.0029) also contributing substantially (**Figure 3L**).

### 3.4 Co-occurrence analysis of disease-specific biomarkers

**Figure 4:**
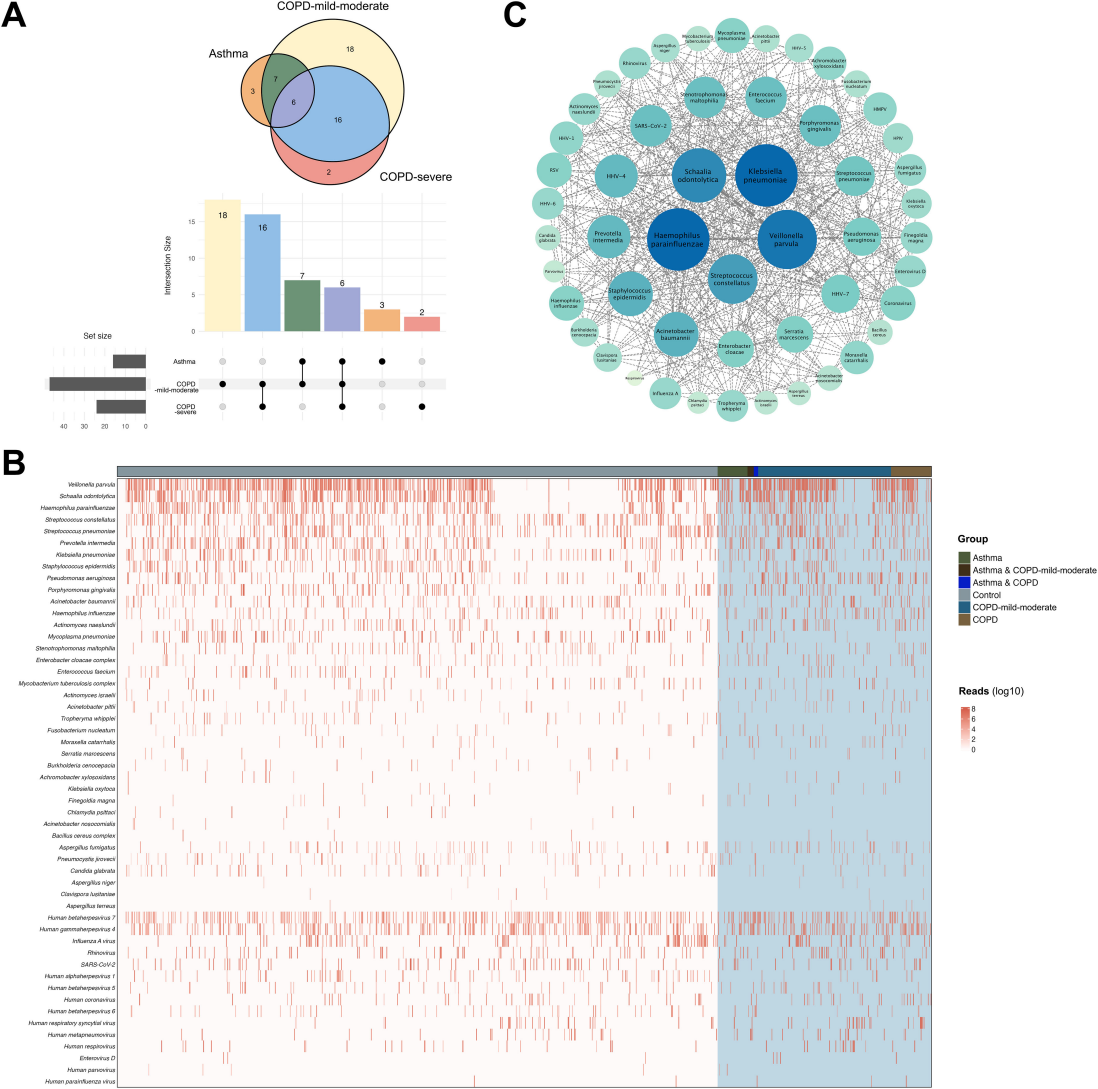
Cross-analysis of infection biomarkers in chronic respiratory diseases. **(A)** Shared and specific biomarkers associated with asthma, COPD-mild-moderate, and COPD-severe. COPD, chronic obstructive pulmonary disease. **(B)** Characterized microorganisms identified by next-generation sequencing (NGS) in all infected patients. The colors above the heat map represent the groups of patients. The depth of color in the heat map represents the number of reads. **(C)** Co-occurrence network of infection biomarkers in chronic respiratory diseases.

In order to delineate both shared and disease-specific microbial signatures among chronic respiratory conditions, a cross-comparison of the top-ranked taxa identified in asthma, COPD-mild-moderate, and COPD-severe patients was performed (**Figure 4A**). The analysis revealed that 6 bacterial taxa were common to all three disease states, including *Schaalia odontolytica*, Human gammaherpesvirus 4, *Actinomyces naeslundii*, *Haemophilus parainfluenzae*, *Pseudomonas aeruginosa*, and Influenza A virus. In contrast, 3 taxa were unique to asthma (including *Prevotella intermedia*, *Finegoldia magna*, and Human parvovirus), 34 were unique to COPD (notably *Veillonella parvula*, Human respiratory syncytial virus, and *Haemophilus influenzae*). Among the 34 microbial signatures, 16 were associated with the COPD-severe (notably Human respiratory syncytial virus, Human coronavirus, and Human parainfluenza virus).

To visualize the abundance patterns of these 52 candidate biomarkers at the patient level, a read-count heat map across all infected individuals was generated (**Figure 4B**). Hierarchical clustering of both taxa and samples segregated patients broadly according to clinical phenotype: asthma patients clustered together driven by elevated reads of *Prevotella intermedia*, *Finegoldia magna*, and Human parvovirus, whereas COPD patients formed distinct clades characterized by higher burdens of *Veillonella parvula*, Human respiratory syncytial virus, and *Haemophilus influenzae*. Moreover, patients classified as having impaired pulmonary function showed a globally higher biomarker load compared to those with preserved function, indicating a potential link between overall microbial burden and pulmonary impairment.

Finally, a co-occurrence network in Cytoscape was constructed to explore ecological interactions among the infection biomarkers (**Figure 4C**). Results showed a rich association (p value of all edges < 0.05) between asthma-related taxa (with *Haemophilus parainfluenzae* and *Schaalia odontolytica* as central hubs) and COPD-related taxa (with *Klebsiella pneumoniae*, *Veillonella parvula*, and *Streptococcus constellatus* exhibiting the highest degree centrality). Together, these findings not only highlight a core set of shared pathogens across chronic airway diseases but also reveal distinctive microbial consortia that may contribute to disease-specific pathogenesis.

## 4 Discussion

In this large-scale cohort study encompassing more than one thousand patients with lower respiratory tract infection, we systematically demonstrated that pulmonary function status and clinical phenotype were accompanied by distinct and highly discriminative microbial signatures. Three principal observations emerged. First, patients with impaired pulmonary function harbored a markedly different airway microbiota composition and a higher total microbial burden than those with preserved function. Second, through machine learning modeling, we identified disease-specific biomarker clusters for asthma, COPD-mild-moderate, and COPD-severe, respectively. Third, co-occurrence analyses revealed intricate interrelationships between biomarkers associated with separate diseases centered on specific microorganisms, highlighting both shared and disease-specific ecological pressures across chronic airway diseases.

Our data extend earlier culture-dependent and 16S rRNA studies that linked impaired spirometry to enrichment of gram-negative pathogens such as *Pseudomonas aeruginosa* (Wölbeling et al., 2010; Faure et al., 2018; Jurado-Martín et al., 2021). We show that this enrichment persists after broad genomic profiling and is accompanied by an expansion of *Veillonella* and *Haemophilus* species in COPD, in line with recent upper-airway sequencing reports (Haldar et al., 2020; Drengenes et al., 2021; Versi et al., 2024). Conversely, we observed selective enrichment of *Schaalia odontolytica* and *Prevotella intermedia* in asthma - organisms rarely captured by conventional diagnostics but increasingly implicated in type-2-high inflammation (Larsen, 2017; Iljazović et al., 2020). The consistent identification of viral taxa such as Human respiratory syncytial virus and Human coronavirus as key predictors of COPD-severe further corroborates mounting virome-centric models of exacerbation pathobiology (Megremis et al., 2023; Rubió et al., 2023).

The observed taxonomic shifts likely reflect several intertwined mechanisms. Structural lung disease and altered mucociliary clearance in COPD favor colonization by facultative anaerobes, while repeated antibiotic exposure and inhaled corticosteroids may create ecological niches for gram-negative opportunists (Zalacaín et al., 1999; Sethi and Murphy, 2001; Desai et al., 2014). In asthma, airway eosinophilia and mucus hypersecretion could enrich oropharyngeal commensals such as *Prevotella* that thrive in low-oxygen, carbohydrate-rich environments (Dunican et al., 2018; Garrido et al., 2024). Importantly, many of the taxa that bridge asthma and COPD (e.g., *Haemophilus parainfluenzae*, Influenza A virus) possess virulence factors capable of disrupting epithelial integrity and potentiating viral-bacterial co-infection, offering a putative mechanistic link between chronic inflammation and acute exacerbation (Veerati et al., 2020; Love and Proud, 2022).

From a translational perspective, the random-forest models illustrate that a minimal microbial feature set can classify airway disease with near-perfect accuracy in silico. If validated prospectively, such models might guide point-of-care molecular diagnostics, enabling rapid differentiation of overlapping clinical phenotypes and allowing tailored antimicrobial or anti-inflammatory therapy. The co-occurrence network further suggests that targeting the newly identified hub organisms - particularly *Haemophilus parainfluenzae* and *Schaalia odontolytica* in the asthma cluster, and *Klebsiella pneumoniae*, *Veillonella parvula*, and *Streptococcus constellatus* in the COPD cluster - could modulate broader pathogenic communities and perhaps attenuate exacerbation risk.

Several important limitations should be acknowledged. First, while strict batch-level negative controls and bioinformatic background subtractions were employed to mitigate reagent and environmental contamination, the inherent challenges of definitively filtering the ‘kitome’ in lower biomass samples cannot be entirely eliminated. Furthermore, our cross-sectional design precludes causal inference, underscoring the need for longitudinal sampling to resolve temporal dynamics and determine whether shifts in key taxa load precede, accompany, or follow clinical exacerbations. The exclusive recruitment of patients from a single tertiary hospital in northeast China may limit generalizability. Independent, geographically diverse cohorts are therefore required to validate our biomarker panels and to assess their incremental predictive value over spirometry and existing clinical scoring systems. Additionally, although patients were stratified based on pulmonary function test criteria, we did not perform a continuous spirometry-specific analysis to determine specific taxa quantitatively associated with lung function parameters (e.g., FEV1 or FVC values). Future studies incorporating such quantitative correlation analyses could provide deeper insights into microbial shifts associated with progressive lung function decline, beyond categorical disease strata. Furthermore, the absence of detailed host immunological data and prior medication history restricted our ability to construct integrated host-microbe models and to adjust for these potential confounders. Mechanistic studies - such as gnotobiotic mouse inoculation with *Schaalia odontolytica* or CRISPR-Cas interrogation of *Haemophilus adhesins* - will be essential to test the hypotheses generated by our co-occurrence network analyses.

In conclusion, this study revealed disease-specific and function-associated microbial architectures across asthma and COPD. These microbial signatures not only discriminated clinical phenotypes with exceptional accuracy but also uncovered interlinked ecological networks that may drive chronic airway pathology. Incorporation of such data into future diagnostic algorithms and interventional trials holds promise for precision medicine in chronic respiratory disease.

## Data Availability

The original contributions presented in the study are included in the article/**Supplementary Material**. Further inquiries can be directed to the corresponding author.

## References

[B1] Blauwkamp T. Thair S. Rosen M. Blair L. Lindner M. Vilfan I. . (2019). Analytical and clinical validation of a microbial cell-free DNA sequencing test for infectious disease. Nat. Microbiol. 4, 663–674. doi: 10.1038/s41564-018-0349-6 30742071

[B2] Desai H. Eschberger K. Wrona C. Grove L. Agrawal A. Grant B. . (2014). Bacterial colonization increases daily symptoms in patients with chronic obstructive pulmonary disease. Ann. Am. Thorac. Soc. 11, 303–309. doi: 10.1513/annalsats.201310-350oc 24423399

[B3] Dicker A. Lonergan M. Keir H. Smith A. Pollock J. Finch S. . (2021). The sputum microbiome and clinical outcomes in patients with bronchiectasis: a prospective observational study. Lancet Respir. Med 9 (8), 885–896. doi: 10.1016/s2213-2600(20)30557-9 33961805

[B4] Dickson R. Schultz M. Van Der Poll T. Schouten L. Falkowski N. Luth J. . (2020). Lung microbiota predict clinical outcomes in critically ill patients. Am. J. Respir. Crit. Care Med 201 (5), 555–563. doi: 10.1164/rccm.201907-1487oc 31973575 PMC7047465

[B5] Drengenes C. Eagan T. Haaland I. Wiker H. Nielsen R. (2021). Exploring protocol bias in airway microbiome studies: one versus two PCR steps and 16S rRNA gene region V3 V4 versus V4. BMC Genomics 22, 3. doi: 10.1186/s12864-020-07252-z 33397283 PMC7784388

[B6] Dunican E. Elicker B. Gierada D. Nagle S. Schiebler M. Newell J. . (2018). Mucus plugs in patients with asthma linked to eosinophilia and airflow obstruction. J. Clin. Invest. 128, 997. doi: 10.1172/jci95693 29400693 PMC5824874

[B7] Evensen A. E. (2010). Management of COPD exacerbations. Am. Fam. Physician 81, 607–613. 20187597

[B8] Faure E. Kwong K. Nguyen D. (2018). Pseudomonas aeruginosa in chronic lung infections: How to adapt within the host? Front. Immunol. 9. doi: 10.3389/fimmu.2018.02416 30405616 PMC6204374

[B9] Feldman C. Shaddock E. (2019). Epidemiology of lower respiratory tract infections in adults. Expert Rev. Respir. Med. 13, 63–77. doi: 10.1080/17476348.2019.1555040 30518278

[B10] Fourgeaud J. Régnault B. Ok V. Da Rocha N. Sitterlé É. Mekouar M. . (2023). Performance of clinical metagenomics in France: a prospective observational study. Lancet Microbe 5 (1), e52–e61. doi: 10.1016/s2666-5247(23)00244-6 38048804

[B11] Frayman K. Armstrong D. Carzino R. Ferkol T. Grimwood K. Storch G. . (2017). The lower airway microbiota in early cystic fibrosis lung disease: a longitudinal analysis. Thorax 72, 1104–1112. doi: 10.1136/thoraxjnl-2016-209279 28280235

[B12] Garrido V. Mukherjee M. Svenningsen S. Nair P. (2024). Eosinophil-mucus interplay in severe asthma: Implications for treatment with biologicals. Allergology Int 73 (3), 351–361. doi: 10.1016/j.alit.2024.03.001 38485545

[B13] GINA (2025). 2025 GINA STRATEGY REPORT: Global Strategy for Asthma Management and Prevention. ( Global Initiative for Asthma (GINA)).

[B14] Gu W. Deng X. Lee M. Sucu Y. Arevalo S. Stryke D. . (2020). Rapid pathogen detection by metagenomic next-generation sequencing of infected body fluids. Nat. Med. 27, 115–124. doi: 10.1038/s41591-020-1105-z 33169017 PMC9020267

[B15] Gu W. Miller S. Chiu C. (2019). Clinical metagenomic next-generation sequencing for pathogen detection. Annu. Rev. Pathol. 14, 319–338. doi: 10.1146/annurev-pathmechdis-012418-012751 30355154 PMC6345613

[B16] Haldar K. George L. Wang Z. Mistry V. Ramsheh M. Free R. . (2020). The sputum microbiome is distinct between COPD and health, independent of smoking history. Respir. Res. 21, 183. doi: 10.1186/s12931-020-01448-3 32664956 PMC7362436

[B17] Iljazović A. Roy U. Gálvez E. Lesker T. Zhao B. Gronow A. . (2020). Perturbation of the gut microbiome by Prevotella spp. enhances host susceptibility to mucosal inflammation. Mucosal Immunol. 14, 113–124. doi: 10.1038/s41385-020-0296-4 32433514 PMC7790746

[B18] Jurado-Martín I. Sainz-Mejías M. McClean S. (2021). Pseudomonas aeruginosa: An audacious pathogen with an adaptable arsenal of virulence factors. Int. J. Mol. Sci. 22, 3128. doi: 10.3390/ijms22063128 33803907 PMC8003266

[B19] Kussek P. Mesa D. Vasconcelos T. Rodrigues L. Krul D. Ibañez H. . (2022). Lower airway microbiota and decreasing lung function in young Brazilian cystic fibrosis patients with pulmonary Staphylococcus and Pseudomonas infection. PloS One 17, e0273453. doi: 10.1371/journal.pone.0273453 36006942 PMC9409528

[B20] Larsen J. (2017). The immune response to Prevotella bacteria in chronic inflammatory disease. Immunology 151, 363–374. doi: 10.1111/imm.12760 28542929 PMC5506432

[B21] Liu J. Ran Z. Wang F. Xin C. Xiong B. Song Z. (2020). Role of pulmonary microorganisms in the development of chronic obstructive pulmonary disease. Crit. Rev. Microbiol. 47, 1–12. doi: 10.1080/1040841x.2020.1830748 33040638

[B22] Love M. Proud D. (2022). Respiratory viral and bacterial exacerbations of COPD—The role of the airway epithelium. Cells 11, 1416. doi: 10.3390/cells11091416 35563722 PMC9099594

[B23] Mayhew D. Devos N. Lambert C. Brown J. Clarke S. Kim V. . (2018). Longitudinal profiling of the lung microbiome in the AERIS study demonstrates repeatability of bacterial and eosinophilic COPD exacerbations. Thorax 73, 422–430. doi: 10.1136/thoraxjnl-2017-210408 29386298 PMC5909767

[B24] Megremis S. Constantinides B. Xepapadaki P. Yap C. Sotiropoulos A. Bachert C. . (2023). Respiratory eukaryotic virome expansion and bacteriophage deficiency characterize childhood asthma. Sci. Rep. 13, 8319. doi: 10.1038/s41598-023-34730-7 37221274 PMC10205716

[B25] Miao Q. Ma Y. Wang Q. Pan J. Zhang Y. Jin W. . (2018). Microbiological diagnostic performance of metagenomic next-generation sequencing when applied to clinical practice. Clin. Infect. Dis. 67, S231–S240. doi: 10.1093/cid/ciy693 30423048

[B26] Niederman M. Torres A. (2022). Respiratory infections. Eur. Respir. Rev. 31, 1133–5. doi: 10.1378/chest.109.5.1133 36261160 PMC9724828

[B27] Rubió J. Megremis S. Pasioti M. Lakoumentas J. Constantinides B. Xepapadaki P. . (2023). Respiratory virome profiles reflect antiviral immune responses. Allergy 78, 1258–1268. 10.1111/all.15634 36595290

[B28] Sethi S. Murphy T. (2001). Bacterial infection in chronic obstructive pulmonary disease in 2000: a state-of-the-art review. Clin. Microbiol. Rev. 14, 336–363. doi: 10.1128/cmr.14.2.336-363.2001 11292642 PMC88978

[B29] Su L. Qiao Y. Luo J. Huang R. Li Z. Zhang H. . (2022). Characteristics of the sputum microbiome in COPD exacerbations and correlations between clinical indices. J. Transl. Med. 20, 76. doi: 10.1186/s12967-022-03278-x 35123490 PMC8818176

[B30] Troeger C. Blacker B. Khalil I. Rao P. Cao J. Zimsen S. . (2018). Estimates of the global, regional, and national morbidity, mortality, and aetiologies of lower respiratory infections in 195 countries, 1990–2016: a systematic analysis for the Global Burden of Disease Study 2016. Lancet Infect. Dis. 18, 1191–1210. doi: 10.1016/s1473-3099(18)30310-4 30243584 PMC6202443

[B31] Veerati P. Troy N. Reid A. Li N. Nichol K. Kaur P. . (2020). Airway epithelial cell immunity is delayed during rhinovirus infection in asthma and COPD. Front. Immunol. 11. doi: 10.3389/fimmu.2020.00974 32499788 PMC7243842

[B32] Versi A. Azim A. Ivan F. Abdel-Aziz M. Bates S. Riley J. . (2024). A severe asthma phenotype of excessive airway Haemophilus influenzae relative abundance associated with sputum neutrophilia. Clin. Transl. Med. 14, e70007. doi: 10.1002/ctm2.70007 39187935 PMC11347389

[B33] Wang Z. Bafadhel M. Haldar K. Spivak A. Mayhew D. Miller B. . (2016). Lung microbiome dynamics in COPD exacerbations. Eur. Respir. J. 47, 1082–1092. doi: 10.1183/13993003.01406-2015 26917613

[B34] Wölbeling F. Munder A. Stanke F. Tümmler B. Baumann U. (2010). Head-out spirometry accurately monitors the course of Pseudomonas aeruginosa lung infection in mice. Respiration 80, 340–346. 10.1159/000319442 20664195

[B35] Yan Z. Chen B. Yang Y. Yi X. Wei M. Ecklu-Mensah G. . (2022). Multi-omics analyses of airway host–microbe interactions in chronic obstructive pulmonary disease identify potential therapeutic interventions. Nat. Microbiol. 7, 1361–1375. doi: 10.1038/s41564-022-01196-8 35995842

[B36] Yu S. Zhang H. Wan L.-P. Xue M. Zhang Y. Gao X. (2022). The association between the respiratory tract microbiome and clinical outcomes in patients with COPD. Microbiol. Res. 266, 127244. doi: 10.1016/j.micres.2022.127244 36335803

[B37] Zalacaín R. Sobradillo V. Amilibia J. Barrón J. Achótegui V. Pijoan J. . (1999). Predisposing factors to bacterial colonization in chronic obstructive pulmonary disease. Eur. Respir. J. 13, 343–348. 10.1034/j.1399-3003.1999.13b21.x 10065679

