# Supplementary material for: Molecular insights into lower respiratory tract microbiota reveal disease-specific biomarkers and shared microbial networks in asthma and COPD
Source: Front Cell Infect Microbiol. 2026 Jul 21;16:1840378. doi: 10.3389/fcimb.2026.1840378 (PMC13433291; doi:10.3389/fcimb.2026.1840378)
Supplement: Supplementary file 1 [file SupplementaryFile1.docx]

Supplementary Material

# 1 Supplementary Figures and Tables

## 1.1 Supplementary Figures

**Supplementary Figure 1.** Workflow of clinical diagnosis. NGS, next-generation sequencing; BALF, bronchoalveolar lavage fluid; CMT, conventional microbiological test.

## 1.2 Supplementary Figures

**Supplementary Figure 2.** Distribution of microorganisms identified by NGS.

## 1.3 Supplementary Figures

**Supplementary Figure 3.** Clinical diagnostic classification of NGS detections in patients with good and poor lung function.

## 1.4 Supplementary Figures

**Supplementary Figure 4.** Number of reads of pathogens detected by next-generation sequencing (NGS) in good and poor lung function groups. (A) For Haemophilus influenzae. The p value was generated by the Mann-Whitney test, with less than 0.05 indicating a statistically significant difference. (B) For Serratia marcescens. (C) For Human betaherpesvirus 6. (D) For Human respiratory syncytial virus.

## 1.5 Supplementary Figures

**Supplementary Figure 5.** Principal coordinate analysis (PCoA) of microorganisms detected by next-generation sequencing (NGS) from patients with good and poor lung function. GLF, good lung function; PLF, poor lung function. The p value less than 0.05 was considered statistically significant. The p values of principal coordinates were generated by the Wilcoxon rank sum test, and the overall p value was generated by the adonis test.

## 1.6 Supplementary Figures

**Supplementary Figure 6.** Optimization of hyperparameters of the random forest models for predicting (A) asthma, (B) COPD-mild-moderate, (C) and COPD-severe. COPD, chronic obstructive pulmonary disease.
